# Gender, planning, and academic expectations in first-year higher education students: testing two alternative mediation models

**DOI:** 10.1186/s41155-020-00142-z

**Published:** 2020-05-07

**Authors:** Sonia Alfonso, António M. Diniz, Manuel Deaño, Fernando Tellado, Mar García-Señorán, Ángeles Conde, Valentín Iglesias-Sarmiento

**Affiliations:** 1 Department of Evolutionary Psychology, Faculty of Education Sciences, University of Vigo, Campus As Lagoas, 32004 Ourense, Spain; 2 Department of Psychology, Research Center in Education and Psychology, School of Social Sciences, Colégio Pedro da Fonseca, PITE - Parque Industrial e Tecnológico de Évora, University of Évora, Rua da Barba Rala, 7000 Évora, Portugal

**Keywords:** College students, Planning, Academic expectations, Gender, Structural equation modeling, Measurement invariance

## Abstract

We examined the relationships among gender, planning, and academic expectations through the testing of two alternative models with latent variables tested with LISREL 8.80: one model considered planning as a mediator of the relationship between gender and academic expectations, and the other model considered academic expectations as mediators of the relationship between gender and planning. Participants were 662 first-year higher-education students from two academic years, predominantly female (60%) and mainly with majors in the juridical-social field (66.2%). The *Inventario sobre Estrategias Metacognitivas* (*IEM*; Inventory of Metacognitive Strategies) and the Academic Perceptions Questionnaire (APQ) were applied at the beginning of the first semester to assess planning and academic expectations, respectively. Multigroup confirmatory factor analysis was used to test the *IEM*’s structure after examining its psychometric properties with the sample from the first academic year (*N* = 338). The test of the alternative mediation models with the full sample indicates that the best model was that with planning as a mediator. In this model, gender directly predicted only two APQ academic expectations, but with the mediation of planning, gender predicted all seven academic expectations. Women showed higher levels of academic expectations and planning than did men. The results are discussed at both the theoretical and practical levels.

## Introduction

It is well known that gender explains in a distinctive way learning-related perceptions and behaviors of higher-education (HE) students, namely, regarding planning and academic expectations (AEs).

Planning has been defined as a hierarchical process that can control the order in which one performs a sequence of operations. The process of controlling one’s actions as a function of purpose requires seeking the problem to be solved, generating and selecting the appropriate strategies to solve it, and executing and checking a sequence of planned actions; in other words, it requires cognitive planning (Das & Misra, 2015). This planning, viewed as an executive function (Best, Miller, & Naglieri, 2011), is linked to the ability to guide behavior by formulating strategies and sequential plans of action and the ability to change plans whenever the situation requires (Naglieri & Otero, 2014).

Through the effect of multiple moderator variables such as cognitive, cultural, and contextual factors, gender differences were observed in HE students’ planning (Voyer & Voyer, 2014; Wang & Degol, 2017): women performed better than men in language, attention, control, and effort in academic tasks, regardless of the area of study (social or mathematical and science), which involves not only a higher level of planning but also academic success.

AEs are defined as a set of representations about what HE students expect to do during their academic life (Deaño et al., 2015) through an interpretation of their HE experiences, in line with past experiences (Cole, Kennedy, & Ben-Avie, 2009). These AEs code HE experiences and academic knowledge (Howard, 2005; Kuh, Gonyea, & Williams, 2005; Soares, Guisande, Diniz, & Almeida, 2006), which, according to the interest and motivations of the students, is recoded and designed for new situations.

Gender differences were observed in AEs. Some studies (e.g., Sax, Bryant, & Harper, 2005) have highlighted a greater emotional dependence of women on their families than of men, which can make it difficult for the former to participate in classroom activities and in interactions with their classmates and teachers. Other studies (Sax & Harper, 2007; Wang & Degol, 2017) have pointed out greater aspirations in women than in men regarding social interaction and involvement in aid actions to others, while men seem to show higher levels of leadership, with higher aspirations to participate in student committees, politics, and association activities. Within a multidimensional view of AEs (Diniz et al., 2018), men, more than women, show aspirations to achieve stable and prestigious future employment, develop autonomy and self-confidence, study abroad, participate in committees, and comply with the expectations of family members regarding the time spent working and career success.

HE students create and execute plans that generate expectations of thinking and action about their goals and the best way to achieve them. Planning regulates students’ thinking and actions about their goals and interests (Rodriguez, 2009; Wang, Spencer, & Xing, 2009). Therefore, AEs can be considered drafts of plans that students confront with reality (Das & Misra, 2015), and based on them, students readjust and modify them, finally consolidating action plans operationalized in manifest conduct. In this sense, planning can be expected to predict the AEs of students when solving problems within the academic context.

Otherwise, cognitive planning as an executive function (Best et al., 2011; Das & Misra, 2015) can be considered a metacognitive mechanism underlying students’ beliefs. These beliefs generate life plans and concrete actions to fulfill goals and interests (Biggs, 1987; Schraw & Moshmam, 1995); thus, AEs can be viewed as predictors of planning.

According to Tinto (1987), students’ expectations about HE calibrate their levels of academic and social commitment to the institution, favoring their integration. In accordance with this position, AEs are considered a set of cognitions and motivations, translated from the perceptions and aspirations that accompany students’ experiences in the academic context (Howard, 2005; Konings, Brand-Gruwel, van Merrienboer, & Broers, 2008).

Accordingly, with this multidimensional conception of AEs, Almeida et al. (2018), Deaño et al. (2015), and Diniz et al. (2018) found seven dimensions of expectations that students bring with them when they reach HE. Students have expectations about training for employment, personal and social development, student mobility, political/citizen involvement, social pressure, the quality of training, and social interaction. These dimensions were obtained through a multigroup confirmatory factor analysis (CFA), and they seem to support a multifaceted and multidimensional concept across gender and nationality (Diniz et al., 2018).

### Current study

All the relationships among the previously mentioned variables, gender, planning, and AEs, are established in the literature. However, to the best of our knowledge, the relationships among these three variables have never been examined altogether.

**Fig. 1:**
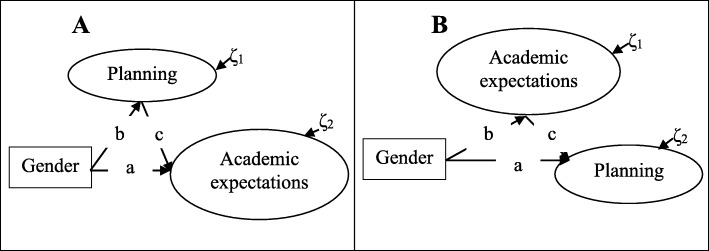
Alternative mediation models. Conceptual diagrams for planning as mediator in **A** and for academic expectations as mediators in **B**. a = direct effect of the observed predictor (OP) on the latent criterion (LC); b = direct effect of the OP on the latent mediator (LM); c = direct effect of the LM on the LC; b × c = indirect effect of the OP on the LC; a + (b × c) = total effect; ζ = structural residuals, random disturbances, or amount of mediators (ζ_1_) and criteria’s (ζ_2_) variance not accounted by predictor(s)

Accordingly, the goal of this study is to examine the relationships among gender, planning, and AEs through the assessment and comparison of the two alternative mediation models with latent variables represented in Fig. 1A, B to choose the more plausible model. Subsidiarily, the test of these models can clarify the nuance of planning being viewed as a predictor of AEs (Rodriguez, 2009; Wang et al., 2009) or AEs being viewed as predictors of planning (Biggs, 1987; Schraw & Moshmam, 1995) found in the literature review.

The use of structural equation modeling (SEM) is appropriate to fulfill this goal, allowing for the simultaneous testing of the relationships among these three variables in a mediation model (Iacobucci, Saldanha, & Deng, 2007). Furthermore, through their examination in the selected model (Fig. 1A, B), it is possible to see if the mediation effect or, in other words, the indirect effect between the observed predictor and the latent criterion makes a significant contribution to the total effect.

## Method

### Participants

A convenience sample of 662 voluntary Spanish first-year HE students (age range = 17–23 years, *Mdn* = 18), mostly composed of women (60.0%, *n* = 397), was used for this study. Participants were selected in two consecutive academic years: sample 2014/15 (*n* = 338, age range = 17–23 years, *Mdn* = 18) and sample 2015/16 (*n* = 324, age range = 17–23 years, *Mdn* = 18), who attended HE for the first time. As a function of the study area, most of the students (66.2%) were enrolled in degrees in the juridical-social field, and the rest were in the scientific-technological field. No association was found between study area and academic years, *χ*^2^(1, *N* = 662) = 1.10, *p* = .295, or between gender and academic years, *χ*^2^(1, *N* = 662) = 3.28, *p* = .073.

### Materials and procedure

Students’ planning was operationalized through the “*Inventario sobre Estrategias Metacognitivas*” (*IEM*; Metacognitive Strategies Inventory; Martínez-Fernández, 2004, 2007), a Spanish adaptation of the Reduced Revised State Metacognitive Inventory (RRSMI; O’Neil & Abedi, 1996). Participants were asked what they do or think when they face a learning activity or problem.

The structure of the 20 *IEM* items, rated on a Likert-type scale ranging from one (*never*) to five (*always*), was examined with a sample of college students through maximum likelihood (ML) exploratory factor analysis (oblique rotation), showing a bifactorial model. These factors, each with 10 items, were named planning (e.g., item 9: “You are aware of the need to plan your course of action”) and self-checking (e.g., item 2: “You check your work while you are doing it”) and presented, respectively, alpha values of .80 and .82. However, the factors presented a very high correlation (.71), and the author did not test an alternative unifactorial model.

Students’ AEs were operationalized using the final version of the “*Cuestionario de Percepciones Académicas (CPA): Versión Expectativas*” (Deaño et al., 2015), named the English Academic Perceptions Questionnaire (APQ; Almeida et al., 2018). The APQ has seven factors, each with six items rated on a Likert-type scale ranging from one (*strongly disagree*) to six (*strongly agree*): training for employment (TE), referring to the conditions of obtaining training to obtain better jobs or enter into the working world (e.g., item 15: “Obtain training to obtain a good job”); personal and social development (PSD), which includes autonomy, self-confidence, critical thinking, and personal improvement through new experiences of academic life (e.g., item 16: “Use academic opportunities to improve my identity, autonomy and self-confidence”); student mobility (SM), linked to the attitude of carrying out part of the studies in international mobility programs, internships or jobs abroad (e.g., item 24: “Obtain international-quality training”); political engagement and citizenship (PEC), which reflects the desire to engage in the political, social, and economic life of the country, to understand how to help improve it, and to participate in specific associative or volunteer activities (e.g., item 25: “Participate in volunteer activities”); social pressure (SP), which includes the items referring to the desire to respond to parents' expectations or to please significant others (e.g., item 5: “Meet my family’s expectations”); quality of education (QE), linked to feeling challenged to deepen one's knowledge and having the personal and material means to stimulate it (e.g., item 13: “Deepen my knowledge of specific subjects”); and social interaction (SI), which includes the will to enjoy some moments of conviviality and fun, dedicating a scheduled weekly time to these activities, different from the study time, which may entail a relationship with classmates (e.g., item 28: “Attend university student parties”).

Regarding the APQ’s psychometric properties, Almeida et al. (2018, Table 2) showed that factors’ convergent validity (CV) and discriminant validity (DV) and its composite reliability (CR) (Fornell & Larcker, 1981) were suitable across countries and genders.

### Procedure

#### Data collection

Students (initial sample, *N* = 669) were selected to ensure the heterogeneity of the major subjects. Data were collected at the beginning of the first semester in the classroom after obtaining teachers’ permission and students’ informed consent. Students who attended HE in previous academic years left the classrooms. The instruments were administered in a counterbalanced way. Seven students were excluded from the sample due to incomplete data (gender variable = 4; *IEM* protocol = 3).

#### Data analysis

IBM SPSS Statistics for Windows (version 21.0) was used for descriptive data analysis, and LISREL 8.80 (Jöreskog & Sörbom, 2006) was used for model estimation and testing.

Given the ordinal categorical nature of the data, analyses were performed using the underlying bivariate normal approach (Jöreskog, 2005). PRELIS 2 (Jöreskog & Sörbom, 1996) produces the polychoric covariance matrix of the underlying latent continuous and normal counterparts of items’ observed responses, the respective asymptotic covariance matrix, and the vector means of the latent responses. They were taken as input for model estimation and testing with the robust Satorra-Bentler (SB) scaled correction (Satorra & Bentler, 1994) in LISREL 8.80 (Jöreskog & Sörbom, 2006) using the SIMPLIS command language (Jöreskog & Sörbom, 1993). Factor measurement units were assigned by fixing the path of one of their items to one.

Models’ fit to the data were examined through the following practical goodness-of-fit (GOF) indices and recommended cutoff values (Hu & Bentler, 1998): the root mean square error of approximation (RMSEA; values close to or below .06), the standardized root mean square residual (SRMR; values close to or below .08), and the comparative fit index (CFI; values close to or above .95). The expected cross validation index (ECVI; Browne & Cudeck, 1993) was also used for the comparison of the two alternative mediation models in Fig. 1; the model with lower ECVI should be chosen.

Following a two-step modeling approach (Jöreskog & Sörbom, 1993), the mediation models presented in Fig. 1 were only tested after the assessment of the structural validity of the *IEM* model.

First, a confirmatory factor analysis (CFA) was performed with the 2014/15 sample. The obtained ML completely standardized estimates allowed for the examination of *IEM* factors’ CV, DV, and CR (Fornell & Larcker, 1981). CV was examined through items’ average variances extracted (AVEs), which should be at least .50. DV was assessed by comparing factors’ shared variance (*φ*^2^ = squared disattenuated correlation) and AVE of each compared factor: DV should be lower than AVE. A factor reliability of .80 is recommended for group comparisons (Nunnally & Bernstein, 1994).

Second, the resulting factorial solution was tested through a multigroup cross-sectional measurement invariance analysis, using both the 2014/2015 and the 2015/2016 samples. It typically starts with the testing of the form-invariant model, where all parameters are freely estimated across groups, followed by the testing of more stringent equality conditions, specifically *weak*, *strong*, and *strict* invariance (the latter compares with the former) (Meredith, 1993). Under weak invariance, factor loadings are equal across groups. Under strong invariance, factor loadings and intercepts (item values corresponding to the zero value of the factor) are equal across groups. To ensure construct comparability across samples, strong invariance is a sufficient criterion. However, to complete the study of measurement invariance, strict invariance was also examined. In strict invariance, factor loadings, intercepts, and residual (item-specific factor plus random error) variances are equal across groups. Finally, a model can also be partially invariant, indicating differential item functioning (Byrne, Shavelson, & Muthén, 1989).

Comparisons between baseline models (with parameters unconstrained for all groups; smaller *df*) and restricted models (with specific parameters constrained to be equal across groups; larger *df*) were based on the difference (Δ) between models’ CFI and, subsidiarily, on GOF statistics. The cutoff value of .01 was used for the ΔCFI results (Cheung & Rensvold, 2002).

Finally, the two alternative mediation models with latent variables (see Fig. 1) were tested using the full sample. After the selection of the model with better ECVI, the following expression, based on the difference between its total and direct effects (unstandardized), was applied: Δ*z* = total effect − direct effect/root square [(*SE*^2^_(total effect)_ + *SE*^2^_(direct effect)_)/2]. If the value of the test statistic Δ*z* was higher than 1.96, *p* < .05, then it means that the contribution of the indirect effect to the total effect was significant.

## Results

With the 2014/15 sample, the test of the *IEM* bifactorial oblique model showed good fit to the empirical data (SB*χ*^2^ = 305.788, *df* = 169, RMSEA = .049, 90% CI [.040, .058], SRMR = .057, CFI = .983). However, substantively, the solution was inacceptable because of the very high shared variance (*φ*^2^ = .85), indicating major DV problems, for example, between-factor collinearity. Thus, the 20 items must be collapsed into a single factor, as should have been done in the *IEM*’s validation study (see Materials and procedure).

This alternative unifactorial model, with slightly worse GOF results than the bifactorial oblique model but more substantively verisimilar, also showed a good fit (SB*χ*^2^ = 318.430, *df* = 170, RMSEA = .051, 90% CI [.042, .060], SRMR = .058, CFI = .981). However, as seen in Table 1 (M1), despite its very good reliability (CR), its VME revealed an excessive lack of CV.

**Table 1:** IEM unifactorial model in the 2014/2015 sample: completely standardized maximum likelihood estimates, average variance extracted, and composite reliability

	Model 1		Model 2	
Item (factor)	*β*	*R*^2^	*β*	*R*^2^
1 (self-checking)	.58	.34	–	–
2 (planning)	.62	.39	.60	.36
3 (planning)	.61	.38	.64	.40
4 (planning)	.57	.32	–	–
5 (self-checking)	.57	.32	–	–
6 (self-checking)	.51	.26	–	–
7 (self-checking)	.51	.26	–	–
8 (planning)	.61	.37	.61	.37
9 (self-checking)	.63	.40	.67	.45
10 (self-checking)	.39	.15	–	–
11 (planning)	.59	.34	–	–
12 (planning)	.60	.36	.60	.36
13 (self-checking)	.69	.48	.69	.47
14 (self-checking)	.66	.43	.65	.42
15 (self-checking)	.57	.33	–	–
16 (planning)	.63	.40	.64	.41
17 (planning)	.56	.31	–	–
18 (self-checking)	.63	.40	.61	.38
19 (planning)	.57	.33	–	–
20 (planning)	.65	.43	.65	.42
AVE	.35		.41	
CR	.91		.87	

*Factor* factor name in the *IEM* bi-factorial oblique model, *β* factor loading, *R*^*2*^ (communality) = 1 − *ε* (standardized residual), *AVE* average variance extracted, *CR* composite reliability

To achieve a more acceptable CV, the 10 items with lower *R*^*2*^ were excluded, and the model (Table 1, M2) fit well to the data (SB*χ*^2^ = 43.235, *df* = 35; RMSEA = .026, 90% CI [.000, .050], SRMR = .040; CFI = .997), with very good CR, as in M1, but still not a good VME. The exclusion of more items could improve the factor’s CV, but achieving the desired AVE value would not be easy (e.g., VME = .43, excluding items 2, 8, 12, and 18). Thus, this 10-item solution represents an acceptable tradeoff between statistical results and factor content heterogeneity. The factor, with six items from the *IEM* bifactorial oblique model related to planning and four items related to self-checking, was named planning, corresponding to a derivation based on theory and empirical results that point out that self-checking is interconnected with planning (Das & Misra, 2015).

This alternative 10-item *IEM* unifactorial model did not show strong invariance, presenting an inadmissible ΔCFI result (Table 2, see also RMSEA). However, the model was partially and strongly invariant across samples because the intercepts of items 2 and 12 were higher in the 2015/16 sample (item 2 = 1.97 vs. 1.32 in the 2014/15 sample; item 12 = 2.73 vs. 1.68 in the 2014/15 sample). Nevertheless, those items belong to the same category of content (Table 1), and using an interpretation in terms of item response theory (Ferrando, 1996), the noninvariance of intercepts does not signal different levels of items ambiguity but simply different levels of item attractiveness between the samples. Therefore, this lack of intercept equivalence did not change the meaning of the factor across samples because weak invariance was achieved. The partially and strongly invariant model had good fit to the empirical data, and partial strict invariance was then tested and achieved (Table 2).

**Table 2:** Measurement invariance of the 10-item IEM unifactorial model across 2014/2015 and 2015/2016 samples

Model	Form invariance	Weak invariance	Strong invariance	Strong invariance^a^	Strict invariance^a^
*df*	70	79	89	87	97
SBχ^2^	105.92	120.36	396.81	134.37	160.01
RMSEA [90% CI]	.039 [.023; .054]	.040 [.025; .054]	.103 [.092; .113]	.041 [.026; .054]	.044 [.032; .056]
SRMR (2014/15)	.040	.049	.051	.048	.059
SRMR (2015/16)	.055	.069	.072	.068	.080
CFI	.990	.989	.917	.987	.983
ΔCFI	–	**.001**	.072	**.002**	**.004**

Results in bold indicate between-samples equivalence

*SB* Satorra-Bentler, *RMSEA* root mean square error of approximation, *SRMR* standardized root mean square residual, *CFI* comparative fit index, *Δ* difference between baseline (smaller *df*) and restricted models (larger *df*)

^a^Partial: items 2 and 12 with intercepts freely estimated across samples

Overall, the psychometric properties of the 10-item *IEM* unifactorial model (Table 1) and its equivalence across samples (Table 2) indicated that it could be used to test the alternative mediation models represented in Fig. 1 using the full sample of this study.

The results of this test showed that the model represented in Fig. 1A was more plausible (better GOF statistics, specifically, a lower ECVI) than its competitor (Table 3) and was the model selected for further analysis.

**Table 3:** Fit indices of the two alternative mediation models

Model	SB*χ*^2^ (*df*)	RMSEA [90% CI]	CFI	SRMR	ECVI
Panel A	3839.326 (1311)	.054 [.052; .056]	.967	.090	6.17
Panel B	4363.638 (1311)	.059 [.057; .061]	.960	.190	6.97

*ECVI* expected cross-validation index, *SB* Satorra-Bentler, *RMSEA* root mean square error of approximation, *SRMR* standardized root mean square residual, *CFI* comparative fit index

**Fig. 2:**
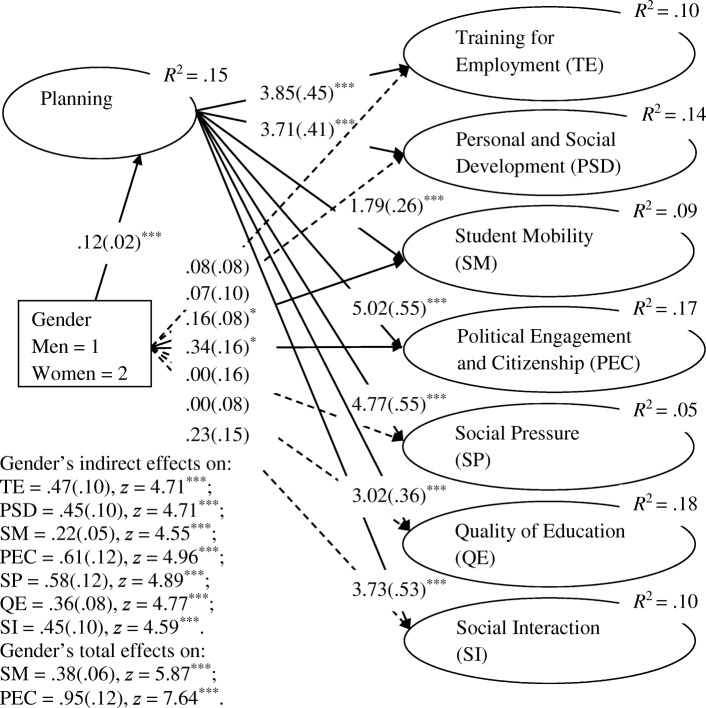
Model of the planning mediation effect on the predictive relationships between gender and academic expectations. Unstandardized robust maximum likelihood estimates for structural relationships. Standard errors are between parentheses. Dashed arrows = non-significant paths. *p < .05. ***p < .001

Figure 2 shows that all the indirect structural paths of the model were statistically significant: women showed higher levels of AEs than men. Interestingly, the gender predictive relationships of TE, PSD, SP, QE, and SI were only observed through the mediation of planning (“complete mediation”; Iacobucci et al., 2007). Moreover, the predictive relation of students’ planning and AEs was weaker for SM and stronger for the other dimensions, especially for PEC and SP. Moreover, according to Cohen’s (1988) taxonomy, the effect size (*R*^2^) of gender on AEs was small on SP; small to medium on SM, TE, and SI; and medium on PSD, PEC, and QE.

Finally, for the two criteria where the direct and indirect effects of gender were simultaneously significant (“partial mediation”; Iacobucci et al., 2007), the results pointed out a difference between the total effect and the direct effect of gender on both SM (Δ*z* = 3.11, *p* = .002) and PEC (Δ*z* = 4.31, *p* < .001). The observed significant differences indicate that the mediation of planning significantly amplified these gender predictive relationships, mainly on PEC.

## Discussion

The literature reviewed in this study was conducted with the following main goal: to test the relationships among gender, planning, and AEs, considering the construct of planning as a predictor of AEs, or vice versa. The result of the testing of two mediation models with gender as a predictor showed that the model represented in Fig. 1A, with planning as a mediator, was more plausible than its competitor, also represented in Fig. 1B, with AEs as mediators. For this reason, the first model was chosen for further analysis.

The significance of all the predictive relations of planning for AEs supports the idea that students who attended HE for the first time create, execute, and regulate plans that generate expectations of thinking and action about their goals and interests (Rodriguez, 2009; Wang et al., 2009) more attached to the AEs of PEC and SP and less to those of SM.

Another factor that could have conditioned the model’s functioning was gender since it is known that in addition to impacting planning (Voyer & Voyer, 2014; Wang & Degol, 2017), it also impacts expectations (Diniz et al., 2018; Sax et al., 2005; Sax & Harper, 2007; Wang & Degol, 2017). In this study, gender had differential direct and mediation effects on planning and AEs. The direct effect on planning, with women showing higher levels of planning than men, has been previously mentioned in the literature (Voyer & Voyer, 2014; Wang & Degol, 2017), but women showed higher levels than men in AEs, and this result is opposed to the one previously found in first-year Spanish and Portuguese students by Diniz et al. (2018), with data collected at the beginning of the second semester: men showed higher levels than women in five out of seven AEs (TE, PSD, SM, PEC, and SP). The authors argued that this result was because women may have adjusted their initial expectations. In addition, planning underlies these AEs’ changes since women present higher levels of self-checking and task planning than do men (Voyer & Voyer, 2014; Wang & Degol, 2017). Another contradictory result is that Diniz et al. (2018) found gender differences in five AEs, whereas in the present study, only the direct effect of gender on SM and PEC was observed. The other AEs (TE, PSD, SP, QE, and SI) were predicted through the mediation of planning (“complete mediation”; Iacobucci et al., 2007).

Overall, these different results lead to the educated guess that the timing when the measurement of AEs occurs (at the beginning of the studies or later on in the second semester) can determine the choice of one or another of the tested mediation models, as well as the differential impact of gender on AEs. In the current study, the best model was the one with planning as a mediator. If another measurement moment had been chosen, the model with AEs as mediators may have been the best because the readjusted students’ beliefs and expectations, driven by life experience in the academic context, could generate new life plans according to the reformulated goals and interests. This is an interesting issue to be further analyzed.

At a theoretical level, this study highlights the relationship between task action goals and first-year HE students’ AEs (Kuh et al., 2005; Pleitz, MacDougall, Terry, Buckley, & Campbell, 2015; Tinto, 1987). It supports the belief that metacognition is a high-level executive function of the general domain, applicable to specific domains, such as AEs (Rodriguez, 2009). At a practical level, interventions focused on action planning and on work checking while doing so may have positive effects not only in the promotion of success in academic tasks (Das & Misra, 2015) but also on AEs, thus facilitating students’ adjustment to their new social and academic context.

Finally, considering the two AEs where the direct and indirect (i.e., through the mediation of planning) effects of gender were simultaneously significant (“partial mediation”; Iacobucci et al., 2007), SM and PEC, the indirect effect amplified the gender impact on them. Strictly speaking, planning accentuates the discrepancy between women (higher levels) and men (lower levels) in these AEs, more notoriously in PEC.

## Conclusions

In line with the results of the current study, intervention programs to enhance academic success in HE should focus on planning at the beginning of studies, especially for men. Furthermore, according to the results of the current study and the study of Diniz et al. (2018), such programs should also address gender differences, focusing on all AEs, especially on PEC in women.

Despite the limitations in external validity due to the nonprobabilistic sampling procedure used in this study (its replication with other samples or, better, with a representative sample is desirable), some aspects related to its internal validity guarantee that the statistical conclusions are reliable: the sample’s dimension, academic major heterogeneity, and the counterbalancing of instruments’ administration. Still related to this study’s internal validity, the instrument used to operationalize planning (*IEM*; Martínez-Fernández, 2004, 2007; O’Neil & Abedi, 1996) was modified at the structural and measurement levels to achieve acceptable structural validity. These changes are justifiable in statistical terms because an appropriate method for the analysis of models with ordinal variables was used: CFA with the SB scaled correction (Jöreskog, 2005), with multigroup measurement invariance testing (Byrne et al., 1989; Cheung & Rensvold, 2002; Jöreskog, 2005; Meredith, 1993), and examination of CV, DV, and CR (Fornell & Larcker, 1981). The observed lack of DV between the *IEM*’s self-checking and planning factors can be explained because planning falls under Das and Misra’s (2015) conception of metacognitive control: self-checking is interconnected with planning.

It is recommended for future research to test the model considering other predictors, such as temporal stability (e.g., beginning/end of the first semester) and sociodemographic characteristics (e.g., nationality). It would also be appropriate to analyze the model’s relationships with academic results and assess cognitive planning with a task resolution battery or through the registration and analysis of conduct, rather than assessing planning through questionnaires. Finally, interventions on students’ cognitive planning are recommended to clarify whether it truly has a positive effect on AEs.

## Data Availability

The datasets used and analyzed during the current study are available from the corresponding author on reasonable request.
